# Despeckle Filtering for Multiscale Amplitude-Modulation Frequency-Modulation (AM-FM) Texture Analysis of Ultrasound Images of the Intima-Media Complex

**DOI:** 10.1155/2014/518414

**Published:** 2014-03-09

**Authors:** C. P. Loizou, V. Murray, M. S. Pattichis, M. Pantziaris, A. N. Nicolaides, C. S. Pattichis

**Affiliations:** ^1^Departement of Computer Science, School of Sciences, Intercollege, 92 Ayias Phylaxeos Street, P. O. Box 51604, CY-3507 Limassol, Cyprus; ^2^Departement of Electrical Engineering, Universidad de Ingenieria y Tecnologia, 2221 Lima, Peru; ^3^Departement of Electrical and Computer Engineering, The University of New Mexico, Albuquerque, NM 87131, USA; ^4^Cyprus Institute of Neurology and Genetics, 1683 Nicosia, Cyprus; ^5^The Vascular Screening and Diagnostic Centre, 1080 Nicosia, Cyprus; ^6^Departement of Computer Science, University of Cyprus, 1678 Nicosia, Cyprus

## Abstract

The intima-media thickness (IMT) of the common carotid artery (CCA) is widely used as an early indicator of cardiovascular disease (CVD). Typically, the IMT grows with age and this is used as a sign of increased risk of CVD. Beyond thickness, there is also clinical interest in identifying how the composition and texture of the intima-media complex (IMC) changed and how these textural changes grow into atherosclerotic plaques that can cause stroke. Clearly though texture analysis of ultrasound images can be greatly affected by speckle noise, our goal here is to develop effective despeckle noise methods that can recover image texture associated with increased rates of atherosclerosis disease. In this study, we perform a comparative evaluation of several despeckle filtering methods, on 100 ultrasound images of the CCA, based on the extracted multiscale Amplitude-Modulation Frequency-Modulation (AM-FM) texture features and visual image quality assessment by two clinical experts. Texture features were extracted from the automatically segmented IMC for three different age groups. The despeckle filters hybrid median and the homogeneous mask area filter showed the best performance by improving the class separation between the three age groups and also yielded significantly improved image quality.

## 1. Introduction

The World Health Organization ranks cardiovascular disease (CVD: coronary artery disease, cerebrovascular disease, and peripheral artery disease) as the third leading cause of death and adult disability in the industrial world [1]. In the United Sates alone, more than 76 million American adults have one or more types of CVD, of whom about half are estimated to be age 65 or older. It is estimated that by 2015, there will be 20 million deaths due to atherosclerosis that will be associated with coronary heart disease and stroke. Atherosclerosis causes enlargement of the arteries and thickening of the artery walls. It begins early in life and silently progresses until clinical events appear.

**Figure 1:**
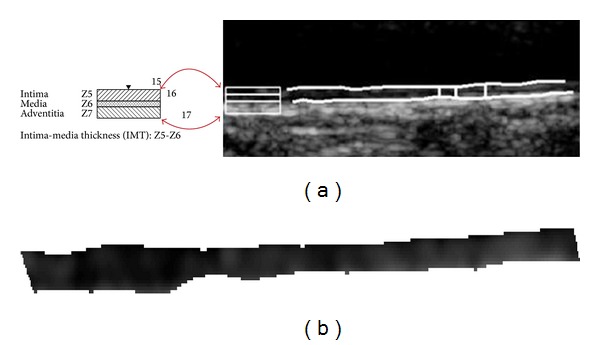
Anatomical locations of the common carotid artery ultrasound image components at the far wall. The IMT is defined as the layer (band) which is comprised by the bands Z5 and Z6 as demonstrated in (a). The intima-media-complex (IMC) in (b) has been extracted using automated segmentation as described in [4, 24], where the IMT_aver_ = 079 mm (between the bands Z5 and Z6, middle bar), IMT_max⁡_ = 0.8367 mm (left bar), IMT_min⁡_ = 0.6356 mm (right bar) and IMT_median_ = 0.75 mm).

The intima-media thickness (IMT) is used as a validated measure for the assessment of atherosclerosis [2, 3] (see Figure 1). We present in Figure 1 anatomical locations of the common carotid artery (CCA) ultrasound image for atherosclerosis indicating the location of the intima-media complex (IMC) at the far wall. The extracted IMC is shown in Figure 1 and has been extracted using an automated snake segmentation algorithm as described in [4]. In [4], we showed that automated IMT, media-layer thickness (MLT), and intima-layer thickness (ILT) measurements could be carried out successfully. It was furthermore proposed that the IMT, its thickness [2, 4], and its textural characteristics [4, 5] may be associated with the risk of developing stroke.

Speckle, a form of locally correlated multiplicative noise, corrupts medical ultrasound imaging making visual observation difficult [6, 7]. Clearly though, excessive despeckling may result in the loss of image structure. Unfortunately, image structure is hard to assess, and this is especially difficult for noisy ultrasound images.

We are interested in characterizing image structure using a multiscale Amplitude-Modulation Frequency-Modulation (AM-FM) texture analysis system as described in [8], and by the independent visual assessment of clinical experts who are asked to judge the quality of the despeckled images. Multiscale AM-FM models represent nonstationary image content using spatially-varying amplitude and phase components. We use the term instantaneous amplitude (IA) to describe spatially-varying amplitude components. Similarly, we use the term of instantaneous frequency (IF) to describe spatially-varying frequency content. AM-FM components are estimated using a multiscale filterbank that is tuned to different frequency bands. Thus, we use the term multiscale AM-FM analysis to summarize the analysis. The promise of AM-FM methods for texture analysis can be summarized in (see [9]) as follows: (i) they provide physically meaningful texture features (e.g., instantaneous frequency in cycles per mm), over multiple scales, at pixel level resolution, (ii) textures can be reconstructed from AM-FM components so that we can visualize content, (iii) we can extract AM-FM decompositions for different frequency coverage, and (iv) we have the recent development of robust methods for AM-FM demodulation (see examples in [9–11]).

Early work in AM-FM image representations has been reported in [12, 13]. In [12], Havlicek et al. discussed the use of multicomponent AM-FM models where Kalman filters are used to track changes in the instantaneous amplitude and instantaneous frequency components. In [13], Havlicek et al. introduced a complex-extension of the Hilbert- transform for images and suggested the use of quasi-eigenfunction approximations (QEAs) for estimating the instantaneous frequency from discrete signals. An early application of AM-FM methods in medical imaging appeared in [14] in electron microscopy. In [15], the authors introduced a foveated video quality assessment method based on the use of continuous-space AM-FM transforms. In [16], the authors extended this research to video compression based on local instantaneous frequency content and the characteristics of the human visual system. An effective model for texture analysis based on multidimensional frequency modulation was introduced in [10]. The multiscale AM-FM methods that are used in this paper were first introduced in [9] and first applied in medical image analysis in [17].

For one-dimensional signals, the empirical mode decomposition pioneered in [18] has been applied to several applications. The empirical mode decomposition uses a special case of AM-FM functions, intrinsic mode functions as basis functions for decomposing signals. These intrinsic mode functions decompose fractional Gaussian noise using a dyadic filter bank as documented in [19]. The popular implementation of the empirical mode decomposition based on [20] requires that the instantaneous amplitude for the first mode be estimated using the extrema of the input signal and then using interpolation to determine the envelope. This approach is clearly sensitive to noise since the envelope estimation is very sensitive to additive noise artefacts. We are not aware of any robust extensions that avoid the significant noise artefacts or robust 2D extensions required for the current application. On the other hand, the use of multiscale AM-FM decomposition allows us to deal with noise through the use of suitable designed band-pass filters [9]. For the current application, the effectiveness of this approach is demonstrated in [8].

While AM-FM decompositions can effectively model nonstationary texture, it is also clear that we want to avoid measuring noise. We want to investigate the use of despeckle filtering for reducing the levels of noise in the image. Yet, excessive despeckling can destroy the nonstationary signal content. It can result in a reduction of the discriminatory power of the texture analysis system introduced in [8, 9, 11], and we expect this to also be detected by the clinical experts as a reduction in image quality. Ultimately, we are interested in the application of despeckle filtering methods that can lead to improvements in computerized texture analysis methods as well as significant improvements in the image quality judged by clinical experts. The use of AM-FM features to characterize the CCA will also provide complimentary information to classical texture analysis features like the gray-scale median, contrast, and coarseness. AM-FM texture features can be associated with the progression of cardiovascular risk for disease and the risk of stroke with age.

Prior research on the use of despeckle filtering on CCA plaque images was reported by Loizou et al. in [21, 22]. In this paper, we investigate the use of two additional despeckle filtering methods, namely, the despeckle filter Kuhawara (see Section 2.3.1, (2)) and the hybrid median filtering method (see Section 2.3.2) and study their effects on image quality and multiscale AM-FM texture analysis on the thin structures of the intima media. As we described in this paper, the new filters gave significantly better results than prior research in [21, 22].

Formally, an input image, *f*(*x*, *y*) is expressed as a sum of AM-FM components using [9, 11] as follows:

(1) $$\begin{array}{c} f\left(x,y\right)=\sum_{n=1}^{N}a_{n}\left(x,y\right)\cos\varphi_{n}\left(x,y\right), \end{array}$$

where *a*_*n*_(*x*, *y*) denotes the *n*th instantaneous amplitude (IA) function, *φ*_*n*_(*x*, *y*) denotes the *n*th instantaneous phase (IP) component, and *n* = 1, 2,…, *N* indexes the different AM-FM components. For each AM-FM component *a*_*n*_(*x*, *y*)cos⁡*ϕ*_*n*_(*x*, *y*), we define the instantaneous frequency (IF) by ∇*ϕ*_*n*_(*x*, *y*) and the magnitude of the IF given by ||∇*ϕ*_*n*_(*x*, *y*)||. Textural characteristics are described in terms of the IA and the IF extracted from different frequency scales. Here, frequency scales are defined based on the IF magnitude and are further classified into low-, medium-, and high-frequency scales.

In [8], AM-FM analysis on the IMC, media-layer (ML), and intima-layer (IL) structures, showed that there are significant differences in AM-FM texture features extracted from different age groups and different sexes. In this paper, we investigate the AM-FM texture features that can show significant differences and also appear to be improving in simulations involving the use of despeckle filtering on ground-truth signals.

The rest of the paper is organized as follows. In the next sections, materials and methods, experimental results, discussion, concluding remarks, and future work are given.

## 2. Materials and Methods

### 2.1. Ultrasound Images Acquisition

A total of 100 B-mode longitudinal ultrasound images of the CCA were recorded using the ATL HDI-3000 ultrasound scanner (Advanced Technology Laboratories, Seattle, USA) [23] as described in [8]. The images were recorded at the Cyprus Institute of Neurology and Genetics in Nicosia, Cyprus. The recordings were carried out in agreement with the Cyprus national bioethics committee rules on clinical trials, and after patient's written consent. For the recordings, we used a linear probe (L74) with a recording frequency of 4–7 MHz [23], a velocity of 1550 m/s, and a1 cycle per pulse, which resulted in a wavelength (spatial pulse length) of 0.22 mm and an axial resolution of 0.11 mm. Furthermore, the scanner is equipped with 64 elements fine pitch high-resolution, 38 mm broadband array, an acoustic aperture of 10 × 8 mm, and a transmission focal range of 0.8–11 cm.

The B-mode scan settings were adjusted to allow for the maximum dynamic range with a linear postprocessing curve. In order to ensure that a linear postprocessing curve is used, these settings were preselected (by selecting the appropriate start-up presets from the software) and were included in the part of the start-up settings of the ultrasound scanner. The position of the probe was adjusted so that the ultrasonic beam was vertical to the artery wall. The time gain compensation (TGC) curve was adjusted, (gently sloping), to produce uniform intensity of echoes on the screen, but it was vertical in the lumen of the artery where attenuation in blood was minimal, so that echogenicity of the far wall was the same as that of the near wall. The overall gain was set so that the appearance of the carotid wall was assessed to be optimal, and slight noise appeared within the lumen. It was then decreased so that at least some areas in the lumen appeared to be free of noise (black). Thus, the standardization effort follows the ACSRS acquisition guidelines as detailed in [24].

Images were acquired with the subject's head rotated by 45° away from the study side. A single longitudinal image was captured at the distal end of the CCA during the diastolic phase of a cardiac cycle. All captured images were revealing optimal visualization of the IMC of the far wall and the near wall of the CCA at the same time, thus corresponding to a midline horizontal longitudinal representation of the CCA walls.

During image acquisition, the sonographers varied spatial resolution to provide optimal imaging at different depths [4, 5]. However, without standardizing image resolution, the estimated AM-FM components would not be comparable. To see this, note that continuous-space image frequencies are expressed in cycles per millimeter, and unless we have a common spatial resolution, the estimated digital frequencies would correspond to different continuous-space (analogue) frequencies. As a result, we then had to use bicubic spline interpolation to resize all digital images to a standard pixel density of 16.66 pixels/mm. The use of bicubic spline interpolation does not add additional information to the image. In other words, interpolation does not recover high-frequency content that was not present in the original acquisition. Thus, when comparing among images, it is important to note that high-frequency content is comparable to the extent that it is shared among all of the resolutions. Also, note that most of the images were acquired at the target resolution. In other words, we only made small corrections to spatial resolution.

The images were also intensity normalized, as described in [25], where a manual selection of blood and adventitia performed by the user of the system is required. The gray-scale intensity normalized image was obtained through algebraic (linear) scaling of the image by linearly adjusting the image so that the median gray level value of the blood was 0–5 and the median gray level of the adventitia (artery wall) was 180–190 [25, 26]. The images were recorded from 42 female and 58 male asymptomatic patients. These subjects had not developed any clinical symptoms, such as a stroke or a transient ischemic attack. The primary-care physicians informed the subjects of our stroke-prevention research study. Overall, patients' ages varied between 26 and 95 years, with a mean age of 54 years. The images were partitioned into three different age groups. In the first group, we included 27 images from patients, who were younger than 50 years old. In the second group, we had 36 patients, who were 50–60 years old. In the third group, we included 37 patients who were older than 60 years.

### 2.2. Simulated Images

**Figure 2:**
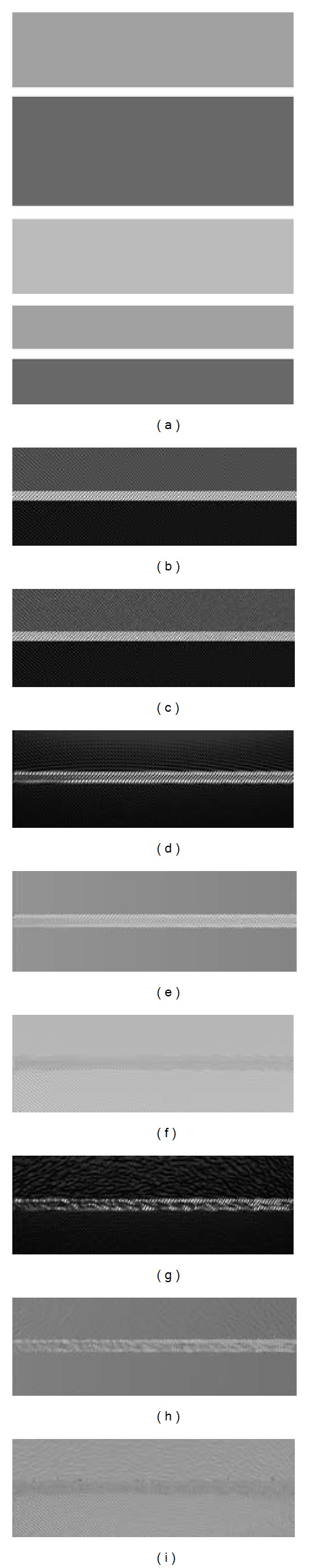
Results using a low frequency scale for the AM-FM methods. (a) Noise-free synthetic IA image. (b) Noise-free synthetic AM-FM image with low frequency information. (c) AM-FM image corrupted with speckle noise. (d) IA estimation from the noisy image of (c). (e) IFx estimation from the noisy image. (f) IFy estimation from the noisy image. (g) IA estimation from the denoised image using the hybrid median filter. (h) IFx estimation from the denoised image using the hybrid median filter. (i) IFy estimation from the denoised image using the hybrid median filter. Under each image we show the same AM-FM results but with a focus (zoom) on the top strip for better visual analysis purposes.

To understand the effects of despeckle filtering on AM-FM estimation, we perform a simulation on a synthetic image (see Figure 2(a)) which was created to resemble clinical ultrasound images. The synthetic IA image was 1024 × 1024 pixels with two strips. For the simulation, we set the IA and the IF as follows:

(2) A(x,y)=158,  π7.5≤ϕx≤π4.5,  ϕy=−ϕx,for  0≤x≤272  (dark  background),A(x,y)=250,  π6.5≤ϕx≤π5.5,  ϕy=ϕx,for  273≤x≤306  (bright  upper  strip),A(x,y)=102,  π7.5≤ϕx≤π4.5,  ϕy=−ϕx,for  307≤x≤702  (dark  background),A(x,y)=250,  π6.5≤ϕx≤π5.5,  ϕy=ϕx,for  703≤x≤750  (bright  lower  stip),A(x,y)=182,  π7.5≤ϕx≤π4.5,  ϕy=−ϕx,for  751≤x≤1023  (dark  backround).

The resulting synthetic image is shown in Figure 2(b). We add multiplicative noise (see Figure 2(c)) to generate *g*_*i*,*j*_ = *f*_*i*,*j*_ + *n*_*i*,*j*_ *f*_*i*,*j*_, where *g*_*i*,*j*_ and *f*_*i*,*j*_ represent the noisy and the original images, respectively, and *n*_*i*,*j*_ is a uniformly distributed random noise with zero mean and noise variance *σ*_*n*_^2^ = 0.07. We show the results after applying a low frequency AM-FM estimation in Figures 2(d)–2(h).  Figure 2(d) illustrates the instantaneous amplitude (IA) estimation from the noisy image, while Figures 2(e)-2(f) show the IF estimation for the *x*- and *y*-directions. In Figure 2(f), we show the IA estimation from the denoised image after using the hybrid median despeckle filter. Finally, we show the IF estimation using this method in Figures 2(g)-2(h).

### 2.3. Despeckle Filtering

#### 2.3.1. Linear Filtering

(1)* First Order Statistics Filtering (DsFlsmv, DsFwiener)*. These filters utilize the first order statistics such as the variance and the mean of a pixel neighbourhood and may be described with a multiplicative noise model [21, 22, 27]. Hence the algorithms in this class may be traced back to the following equation:

(3) $$\begin{array}{c} f_{i,j}=\overset{-}{g}+k_{i,j}\left(g_{i,j}-\overset{-}{g}\right), \end{array}$$

where *f*_*i*,*j*_ is the estimated noise-free pixel value, *g*_*i*,*j*_ is the noisy pixel value in the moving window, $\overset{-}{g}$ is the local mean value of a 5 × 5 rectangular region surrounding and including pixel *g*_*i*,*j*_, *k*_*i*,*j*_ is a weighting factor, with *k* ∈ [0 ⋯ 1], and *i*, *j* are the pixel coordinates. The factor *k*_*i*,*j*_ is a function of the local statistics in a moving window and can be found in the literature [21, 22] as

(4) $$\begin{array}{c} k_{i,j}=\frac{1-{\overset{-}{g}}^{2}\sigma^{2}}{\sigma^{2}\left(1+\sigma_{n}^{2}\right)}. \end{array}$$

The values *σ* ^2^ and *σ*_*n*_^2^ represent the variance in the moving window and the variance of noise in the whole image, respectively. The noise variance is calculated in the logarithmically compressed image, using the average noise variance over a number of windows with dimensions considerably larger than the filtering window [21, 22]. The Wiener filter uses a pixel-wise adaptive method [6, 7, 22, 28] and is implemented as given in (3) with a different weighting factor *k*_*i*,*j*_ = (*σ* ^2^ − *σ*_*n*_^2^)/*σ* ^2^ [13]. For both despeckle filters, which are proposed in this subsection, the moving window size was 5 × 5 and the number of iterations was set to two.

(2)* Homogeneous Mask Area Filtering (DsFkuhawara, DsFlsminsc)*. The Kuhawara despeckle filter is a 1D filter operating in a 5 × 5 pixel neighbourhood by searching for the most homogenous neighbourhood area around each pixel [22, 29]. The middle pixel of the 1 × 5 neighbourhood is then substituted with the median gray level of the 1 × 5 mask. The filter was iteratively applied 2 times on the image.

The DsFlsminsc is a 2D filter operating in a 5 × 5 pixel neighbourhood by searching for the most homogenous neighbourhood area around each pixel, using a 3 × 3 subset window [21, 22]. The middle pixel of the 5 × 5 neighbourhood is substituted with the average gray level of the 3 × 3 mask with the smallest speckle index, *C*, where *C* for log-compressed images is given by

(5) $$\begin{array}{c} C=\frac{\sigma_{s}^{2}}{{\overset{-}{g}}_{s}}, \end{array}$$

where *σ*_*s*_^2^ and ${\overset{-}{g}}_{s}$ represent the variance and mean of the 3 × 3 window. The window with the smallest *C* is the most homogenous semiwindow, which, presumably, does not contain any edge. The filter is applied iteratively one time in the image.

#### 2.3.2. Nonlinear Filtering (DsFmedian, DsFhybridmedian)

The first filter proposed in this subsection [22] is a median filter applied over windows of size 5 × 5. This is extended in the hybrid median despeckle filter, [30] which produces the average of the outputs generated by median filtering with three different windows (cross shape window, ×-shape window, and normal window).

#### 2.3.3. Diffusion Filtering (DsFsrad, DsFnldif)

(1)* Speckle Reducing Anisotropic Diffusion Filtering*. Speckle reducing anisotropic diffusion is described in [7]. It is based on setting the conduction coefficient in the diffusion equation using the local image gradient and the image Laplacian. The speckle reducing anisotropic diffusion filter [7] uses two seemingly different methods, namely, the Lee [27] and the Frost diffusion filters [28]. A more general updated function for the output image by extending the PDE versions of the despeckle filter is [7, 22]

(6) $$\begin{array}{c} f_{i,j}=g_{i,j}+\frac{1}{\eta_{s}}\mathrm{div}\left(c_{\text{srad}}\left(\left|\nabla g\right|\right)\nabla g_{i,j}\right). \end{array}$$

The diffusion coefficient for the speckle anisotropic diffusion, *c*_srad_(|∇*g*|), is derived [7] as

(7) $$\begin{array}{c} c_{\text{srad}}^{2}\left(\left|\nabla g\right|\right)=\frac{\left(1/2\right){\left|\nabla g_{i,j}\right|}^{2}-\left(1/16\right){\left(\nabla^{2}g_{i,j}\right)}^{2}}{{\left(g_{i,j}+\left(1/4\right)\nabla^{2}g_{i,j}\right)}^{2}}. \end{array}$$

It is required that *c*_srad_(|∇*g*|) ≥ 0. The above instantaneous coefficient of variation combines a normalized gradient magnitude operator and a normalized Laplacian operator to act like an edge detector for speckle images. High-relative gradient magnitude and low-relative Laplacian indicate an edge. The filter proposed in this subsection utilizes speckle reducing anisotropic diffusion after (5) with the diffusion coefficient *c*_srad_(|∇*g*|) in (7) [7].

(2)* Coherent Nonlinear Anisotropic Diffusion Filtering*. This filter extends the conduction coefficient using a symmetric positive semidefinite diffusion tensor [31] with the parameters as given in [22]. Therefore, the filter will take the following form:

(8) $$\begin{array}{c} \frac{dg_{i,j,t}}{dt}=\mathrm{div}\left[D\nabla g\right], \end{array}$$

where *D* ∈ *ℜ* ^2*x*2^ is a symmetric positive semidefinite diffusion tensor representing the required diffusion in both gradient and contour directions, and hence enhancing coherent structures as well as edges. The design of *D* as well as the derivation of the coherent nonlinear anisotropic diffusion model may be found in [31] and is given as

(9) $$\begin{array}{c} D=\left(\begin{array}{cc} \omega_{1} & \omega_{2} \end{array}\right)\left(\begin{array}{cc} \lambda_{1} & 0\\ 0 & \lambda_{2} \end{array}\right)\left(\begin{array}{c} \omega_{1}^{T}\\ \omega_{2}^{T} \end{array}\right) \end{array}$$

with

(10) $$\begin{array}{c} \lambda_{1}=\{\begin{array}{cc} \alpha \left(1-\frac{{\left(\mu_{1}-\mu_{2}\right)}^{2}}{s^{2}}\right), & \text{if}\;{\left(\lambda_{1}-\lambda_{2}\right)}^{2}\leq s^{2},\\ 0, & \text{otherwise}, \end{array} \end{array}$$

(11) $$\begin{array}{c} \lambda_{2}=\alpha , \end{array}$$

where the eigenvectors *ω*_1_, *ω*_2_ and the eigenvalues *λ*_1_, *λ*_2_ correspond to the directions of maximum and minimum variations and the strength of these variations, respectively. The flow at each point is affected by the local coherence, which is measured by (*μ*_1_ − *μ*_2_) in (10). The parameters used in this work for the coherent nonlinear anisotropic diffusion filter were *s* ^2^ = 2 and *α* = 0.9, which were used for the calculation of the diffusion tensor *D*, and the parameter step size *m* = 0.2, which defined the number of diffusion steps performed. The local coherence is close to zero in very noisy regions and diffusion becomes isotropic (*μ*_1_ = *μ*_2_ = *α* = 0.9), whereas in regions with lower speckle noise, the local coherence corresponds to (*μ*_1_ − *μ*_2_)^2^ > *s* ^2^ [31].

### 2.4. IMC Snakes Segmentation

All images were automatically segmented to identify the IMC regions. Automatic segmentation was carried out after image normalization and despeckle filtering using the snakes segmentation system proposed and evaluated on ultrasound images of the CCA in [4]. The segmentation system is based on the Greedy active contour algorithm [32]. Using the definitions given in Figure 1, we first segment the IMC [33] by extracting the I5 (lumen-intima interface) and the I7 boundaries (media-adventitia interface). In order to achieve standardization in extracting the thickness from the IMC segments with similar dimensions, the following procedure was carried out. A region of interest of 9.6 mm (160 pixels) in length was first extracted. This was done by estimating the center of the IMC area and then selecting 4.8 mm (80 pixels) left and 4.8 mm (80 pixels) right of the center of the segmented IMC. Selection of the same IMC length from each image is important in order to be able to extract comparable measurements between images and subject groups.

We note that there was no significant difference between the manual and automated segmentation measurements for the IMC [4].

### 2.5. Texture Analysis Using Multiscale Amplitude-Modulation Frequency-Modulation (AM-FM) Methods

Multiscale AM-FM texture features were extracted over different channel filters. We refer to [8] for full details on the approach. Here, we provide a brief summary for completeness.

First, a complex valued image is obtained using an extended 2D Hilbert operator. The operator is implemented by taking the 2D FFT of the input image, zeroing out the upper two frequency quadrants, multiplying the remaining frequency components by 2, and taking the inverse 2D FFT.

The complex-valued output image is processed through a collection of 2D channel filters with passbands restricted over the (nonzeroed) lower two quadrants. We refer to [8] for a clear description of the filterbank. Here, we simply note that we have low-, medium-, and high-frequency scales based on the passband frequency magnitudes. Based on the dyadic frequency decomposition, we have (1) low-frequency components from 1.04 to 2.95 cycles/mm that correspond to instantaneous wavelengths (IWs) from 5.66 to 16 pixels (0.34–0.96 mm); (2) medium-frequency components from 2.08 to 5.89 cycles/mm that correspond to IW from 2.83 to 8 pixels (0.17–0.48 mm); and (3) high-frequency components from 4.17 to 11.79 cycles/mm that correspond to IW from 1.41 to 4 pixels (0.085–0.24 mm) [8].

AM-FM demodulation is carried out separately for the low-, medium-, and high-frequency scales. Adaptively, for each frequency-scale, at each image pixel, we estimate IA by taking the absolute value of the channel response. Then, at each pixel, among the channel responses of each scale, we select the channel that gives the maximum IA. The phase for each scale is then estimated by taking the phase response of the dominant channel.

An adaptive method is used for estimating IF components. The IF components are estimated using

(12) $$\begin{array}{c} \frac{d\phi \left(x,y\right)}{dx}≅\frac{1}{n}\text{arccos}\left(\frac{\overset{-}{f}\left(x+n,y\right)+\overset{-}{f}\left(x-n,y\right)}{2\overset{-}{f}\left(x,y\right)}\right) \end{array}$$

and similarly for *dφ*/*dy*, where $\overset{-}{f}$ denotes the estimated FM image cos⁡*φ*(*x*, *y*)cos⁡*φ*(*x*, *y*). In (12), we consider *n* = 1, 2, 3, 4 for the low frequencies, *n* = 1,2 for the medium frequencies, and *n* = 1 for the high frequencies. Among the IF estimates, we select the one that generates the minimum argument to the arccos function. This is expected to be the most accurate [11]. The AM-FM texture features are then formed by taking the 32-bin histograms of the resulting IA and IF estimates from each one of the three frequency scales.

The Mann-Whitney rank sum test (for independent samples of different sizes) [34] was used in order to identify if there were significant differences (S) or not (NS) between the extracted AM-FM texture features at *P* < 0.05. The results will be explained in Section 3.2 and summarized in Table 3.

### 2.6. Visual Evaluation by Experts

The visual evaluation was carried out according to the ITU-R recommendations with the Double Stimulus Continuous Quality Scale (DSCQS) procedure [21, 22]. The 100 segmented IMC structures of the CCA were evaluated visually by two vascular experts, a cardiovascular surgeon, and a neurovascular specialist before and after despeckle filtering. For each case, the original and the despeckled images were presented at random and without labeling to the two experts. The experts were asked to assign a score in the one to five scale corresponding to low and high subjective visual perception criteria. Five was given to an image with the best visual quality. Therefore, the maximum score for a filter is 500, if the expert assigned the score of five for all the 100 images. For each filter, the score was divided by five to be expressed in percentage format. The experts were allowed to give equal scores to more than one image in each case. For each class and for each filter the average score was computed.

We have, furthermore, used the recently proposed NIQE index assessment tool [35] for objective evaluation of the quality of the images. The tool is based on the construction of a quality aware collection of statistical features based on a simple and successful space domain natural scene statistic model. These features are derived from a collection of natural, undistorted images. The quality of the despeckled image is expressed as a simple distance metric between the model statistics and those of the original image. A software release of the NIQE index is available at http://live.ece.utexas.edu/research/Quality/index.htm.

## 3. Experimental Results

### 3.1. Artificial Carotid Image

Despeckle filtering was evaluated on an artificial carotid artery image corrupted by speckle noise (see Figure 2(a)) as described in the materials and methods section.  Figure 2 presents the results using a low frequency scale for the AM-FM methods. In Figure 2(a), we present the original synthetic image, while in Figures 2(b) and 2(c), we show the original synthetic image with low frequency information and the image from Figure 2(b) with speckle noise, respectively. In Figure 2(d) we show the IA estimation from the noisy image, while in Figure 2(e), we present the IFx estimation from the noisy synthetic image. In Figure 2(f), the IFy estimation from the noisy synthetic image is illustrated while in Figure 2(g), the IA estimation from the despeckled image using the hybrid median filter is shown. Finally in Figures 2(h) and 2(i), we present the IFx estimation from the despeckled artificial image using the hybrid median filter and the IFy estimation from the despeckled image using the hybrid median filter, respectively. Below each figure we present the zoom of the top part of the synthetic image AM-FM results including the top strip for visualization purposes.

Table 1 presents the results of despeckle filtering demonstrating its advantages applied to a synthetic AM-FM example. We note significant noise estimation improvements for the narrow strip for both the IF component for both the *x*- and *y*-direction. Here, we were not interested in the IA error since it was piecewise-constant and estimation could be significantly improved by simply using median-filtering on the estimated values. The results are reported over the low-frequency scales where most of the image energy is usually concentrated.

### 3.2. Real Carotid Ultrasound Images

**Figure 3:**
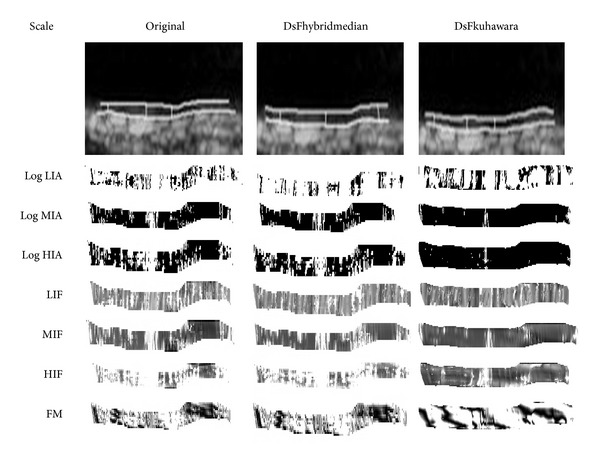
AM-FM analysis of the IMC original (1st column) and despeckled images with the DsFhybrimedian (2nd column) and DsFkuhawara (3rd column), from a male asymptomatic subject aged 49. In the 1st row, the IMT measurements of the original (IMT_aver_ = 0.66 mm, IMT_max⁡_ = 0.827 mm, IMT_min⁡_ = 0.526 mm, IMT_median_ = 0.68), DsFhybrimedian (IMT_aver_ = 0.69 mm, IMT_max⁡_ = 0.91 mm, IMT_min⁡_ = 0.53 mm, IMT_median_ = 0.69), and DsFkuhawara (IMT_aver_ = 0.63 mm, IMT_max⁡_ = 0.77 mm, IMT_min⁡_ = 0.49 mm, IMT_median_ = 0.63 mm) are shown. In the following rows we present the AM-FM components of the instantaneous amplitude of Log of LIA, MIA, and HIA, and of instantaneous frequency of LIF, MIF, and HIF. The last row shows the FM demodulation (integral of the IF) of the images in the low frequencies. For better visualization, the images have been interpolated to be 300 × 20 pixels.

We show in Figure 3 an example of the original IMC ultrasound image in the first column and the corresponding despeckled images with hybrid median, and Kuhawara filters in the second and third columns, respectively. The figure also shows the logarithmic views of the IA components LIA, MIA, and HIA; the IF components LIF, MIF, HIF; and the reconstructed FM component. The last row shows the FM demodulation (integral of the IF) of the images in the low frequencies. For better visualization, the images have been interpolated to be 300 × 20 pixels. In this Figure, image regions where the estimated instantaneous frequency is outside the low-scale frequency range are depicted as dark (black). By comparing the figures (in the three different columns of Figure 3), it is clear that the hybrid median approach has improved the estimation significantly. In other words, there are fewer dark regions in the results of the second column than there are in the third column of Figure 3 (see Log LIA column). For the Kuhawara filter (see Figure 3, third column), segmentation gave a slightly expanded version of the original segmentation results. Furthermore, the area of the dark regions appears to be greater than that for the hybrid median filter. Also, in this case, the Kuhawara filter does not show significant improvements over the results on the original image.

The first part of Table 2 tabulates the results of the visual evaluation of the original and despeckled IMC images made by two experts, a cardiovascular surgeon and a neurovascular specialist. It is clearly shown in Table 1 that the best despeckle filter is the hybrid median with a score of 73%, followed by Kuhawara with a score of 71%. It is interesting to note that these two filters were scored with the highest evaluation markings by both experts. The other filters gave poorer performance, like the DsFnldif, DsFlsminsc, and DsFsrad, that gave an evaluation score of 62%, 58%, and 56%, respectively. The third row of Table 2 presents the overall average percentage (%) score assigned by both experts for each filter. The second part of Table 2 illustrates the objective evaluation performed, for all despeckled filters investigated, between the original and the despeckled images by using the NIQE index. It is shown that the best results were obtained by the hybrid median despeckle filter (NIQE = 0.987) followed by the Kuwahara (NIQE = 0.981) despeckle filter. The last row of Table 2 presents the final filter ranking.

Table 3 presents the statistical analysis between the Low, Medium, and High AM-FM features extracted from the IMC for the three different age groups, below 50 (<50), between 50 and 60 (50–60), and above 60 (>60) years old based on the Mann-Whitney rank sum test, showing only the features that exhibited statistically significant difference at *P* < 0.05. It is shown in Table 3 that all the despeckle filters investigated increased the number of AM-FM features that exhibited significant differences between the different ages (compare the first row for the Original images versus the rest of the columns that represent the despeckled images). More specifically, using the hybrid median filter, we can use the following AM-FM components that demonstrated significant differences for differentiating between the different IMC age groups.

- For the <50 and 50–60 years old, use the LIA component.
- For the <50 and >60 years old, use the MIA, and/or the LIF, and the HIF components.
- For the 50–60 and >60 years old, use the LIA, and/or the LIF, and the MIF components.

Also, the Kuhawara despeckle filter that can be used showed a similar performance as above, except for the LIF component in (c).

Table 4 presents a comparison of the mean, standard deviation (STD), median, 5%, 10%, 25%, 75%, 90%, and 95% quartiles between the high, medium, and low AM-FM features extracted from the IMC for the original and the despeckled filters DsFhybrimedian and DsFkuhawara for the three different age groups, below 50 (<50), between 50 and 60 (50–60), and above 60 (>60) years old. Only those features that showed significant differences in almost all different age groups according to Table 3 are presented. The results indicate that for the high instantaneous frequency (HIF) magnitude median for the IMC, the 75th percentile value of the >60 age group remains lower than the median value of the <50 age group (cycles/mm). Furthermore, we note the original HIF standard deviation of 0.028 (<50) and 0.016 (>60) cycles/mm versus the hybrid median filter with 0.1776 (<50) and 0.126 (>60) cycles/mm and the Kuhawara filter with 0.178 (<50) and 0.125 (>60) cycles/mm. It is clear that image despeckling produces more than a 5-fold increase in the spread of the high instantaneous frequency range. This suggests that high-frequency texture information does benefit from despeckling.

This is a positive result since speckle noise can have detrimental effects on high frequencies. Another significant difference is observed in the standard deviation for the Low Instantaneous Frequency (LIF). In this case, it is interesting to compare the LIF for 50–60 that gives 0.038 cycles/mm for the despeckled images versus 0.058 cycles/mm for the hybrid median filter. This shows a significant increase in the low-frequency magnitude spread in the results for the hybrid median filter. As discussed in Figure 2, successful AM-FM estimation over larger regions of the image also contribute to this larger spread. On the other hand, note a somewhat smaller spread for the >60 group (0.052 versus 0.047 cycles/mm). Overall, it is clear that the IF spreads for the despeckled images tend to either have a significant increase or remain essentially the same as the original (speckled) images.

## 4. Discussion and Concluding Remarks

Clinically, no significant changes are anticipated in the IMT before the age of 50 [36]. It was shown in [4] (based on a similar group of subjects with the one used in this study as well) that between the ages of 50 and 60, the age borderline for the young (<50 years) and the adult (>60 years) ages and a subtle increase in IMT can be demonstrated and IMC textural changes can be initially observed. Above the age of 60, IMT increases and changes in the IMC are more evident. Moreover, most of the stroke incidences in this age group are associated with the carotid atherosclerosis disease. Significant texture changes between the different age groups were reported in [5] for age and sex. More specifically: (a) some of the texture features can be associated with the increase (difference variance, entropy) or decrease (grey scale median (GSM)) of patient's age, (b) the GSM of the media layer (ML) falls linearly with increasing ML thickness (MLT) and with increasing age, (c) the GSM of male subjects is larger than that of female subjects (see Figure 4), and (d) male and female subjects may be better distinguished using texture features extracted from the IMC.

Despeckle filtering improved the class separation between the three age groups as measured by the number of significantly different AM-FM texture features. The improvements were also reflected in better instantaneous frequency estimation and also the significantly improved image quality as evaluated by two clinical experts. In terms of performance, the nonlinear hybrid median despeckle filter (DsFhybridmedian) gave the best results, followed by the homogeneous mask area filter (DsFkuhawara). More specifically, using the hybrid median filter, we can use the following AM-FM components that demonstrated significant differences for differentiating between the different IMC age groups: (a) for the <50 and 50–60 years old use the LIA component; (b) for the <50 and >60 years old use the MIA, and/or the LIF, and the HIF components; (c) for the 50–60 and >60 years old use the LIA, and/or the LIF, and the MIF components. Also, the Kuhawara filter that can be used showed a similar performance as above, except for the LIF component in (c).

These filters combined with multiscale AM-FM analysis can be used to differentiate between the three age groups investigated (that could loosely correspond to low, medium, and high-risk). It should be noted that this is not the case when the nondespeckled AM-FM analysis was used as documented in [8] (and shown in Table 3). In fact, almost all despeckling filters improved class separation over the nondespeckled filters. In Table 3, this is reflected in the increased number of significant (despeckled) AM-FM features that can be used to differentiate between classes.

The intensity normalization method used in this study was found to be helpful in the manual contour extraction [22, 26] as well as the snakes segmentation of the IMC [4, 33] and the extraction and evaluation of texture features from ultrasound images of the CCA [5]. The method uses prior knowledge of the high- and low-intensity values of the adventitia and blood so that the new intensity histogram of the lesion has its maximum peak close to its average gray-scale value [25]. Moreover, this method increased the classification accuracy of different plaque types as assessed by the experts [37]. Ultrasound image normalization was carried out prior to segmentation of the IMT on carotid artery ultrasound images for increasing the image contrast in [38]. Using the above intensity normalisation method, the AM-FM texture analysis proposed in this study may be also applied directly to the logarithmic compressed images.

The proposed despeckle filtering methods have been evaluated on 550 ultrasound images of the CCA together with other despeckle filters in [21, 22, 26] using texture features, image quality metrics, observers evaluation, and kNN classification. More specifically, it was shown that these filters can be used to improve the class separation between asymptomatic and symptomatic subjects based on the statistics of the extracted texture features and improve the classification success rate and the visual evaluation by experts. A number of other despeckle filtering methods have been proposed by other researches in the last 20 years, for increasing the accuracy of edge detection in images [39], improve the image visual perception evaluation [7, 21, 22, 27–31], and aid the segmentation of the IMC and atherosclerotic carotid plaque in ultrasound images [4, 33] or videos of the CCA [40]. Recently, a despeckle filtering toolbox for ultrasound videos have been proposed [41], which can also be downloaded in executable code from http://www.medinfo.cs.ucy.ac.cy/.

Future work will investigate whether it is possible to identify a group of patients at risk of atherosclerosis based on their texture features extracted from the IL, ML, and the IMC of high-resolution ultrasound images of the CCA. It may also be possible to identify and differentiate those individuals into high and low risk groups according to their cardiovascular risk before the development of plaques. The proposed methodology may also be applied to a group of people, which already developed plaques in order to study the contribution of the ML texture features to cardiovascular risk. Both groups of patients may benefit by prognosing and managing future cardiovascular events. Another possible future application of the proposed methodology is that it can be used to investigate possible effects of statins or other drugs in texture feature changes of the ML of the CCA.

The results will need to be validated on larger datasets before they can be transitioned to clinical use. Furthermore, the effect of despeckling on automated segmentation, texture analysis, and classification of atherosclerotic plaques needs to be further researched.

## Figures and Tables

**Table 1:** Despeckle filtering demonstrating its advantages applied to a synthetic AM-FM example (see text for details). Note significant noise estimation improvements for the narrow strips.

Frequency component	*x*-component of low instantaneous frequency (LIF*x*)			*y*-component of low instantaneous frequency (LIF*y*)		
	Backgrounds	Strips	Combined	Backgrounds	Strips	Combined
Noise-free, low-scale AM-FM (upper bound of what can be achieved)	3.9*E* − 06	7.6*E* − 02	2.7*E* − 03	2.9*E* − 02	5.5*E* − 01	4.8*E* − 02
Speckled image, low-scale AM-FM estimation (no despeckling)	5.5*E* − 04	1.2*E* − 01	4.9*E* − 03	3.3*E* − 02	4.9*E* − 01	4.9*E* − 02
Despeckling using DsFlsmv	7.3*E* − 04	5.1*E* − 02	2.5*E* − 03	6.8*E* − 02	2.6*E* − 01	7.5*E* − 02
Despeckling using DsFhybridmedian	1.6*E* − 03	4.6*E* − 02	3.1*E* − 03	6.2*E* − 02	4.1*E* − 01	7.4*E* − 02
Despeckling using DsFKuhawara	6.9*E* − 03	7.3*E* − 02	9.3*E* − 03	6.4*E* − 02	4.8*E* − 01	7.9*E* − 02

**Table 2:** Percentage scoring of visual and objective evaluation of the original and despeckled images by the experts and the natural image quality evaluation (NIQE) index. Bolded values show best performance.

Experts	original	First order statistics		Homogeneous mask area		Non-linear filtering		Diffusion	
		DsFlsmv	DsFwiener	DsFkuhawara	DsFlsminsc	DsFmedian	DsFhybridmedian	DsFnldif	DsFsrad
Visual Evaluation									
Expert 1	33	26	27	**65**	51	43	**71**	59	61
Expert 2	40	30	23	**77**	65	47	**75**	65	51

Average %	37	28	25	**71**	58	45	**73**	62	56

Objective Evaluation									
NIQE	0.861	0.834	0.810	**0.981**	0.956	0.901	**0.987**	0.962	0.923
Ranking	7th	8th	9th	**2nd **	4th	6th	**1st **	3rd	5th

NIQE: Naturalness image quality evaluation.

**Table 3:** Statistical analysis between the low, medium and high AM-FM features extracted from the IMC for the automated segmentation measurements for the three different age groups, below 50 (<50), between 50 and 60 (50–60), and above 60 (>60) years old based on the Mann-Whitney rank sum test for all despeckle filtering techniques. Only the features that exhibited statistical significant differences at *P* < 0.05 are shown.

Filter name	Age groups	50–60	>60	Score	Table 2 ranking
Original (see also) [8]	<50		MIA	3	7th
	50–60		LIA/HIF		

DsFlsmv	<50	MIA/HIF	MIA/LIF	5	8th
	50–60		MIF (0.4)		

DsFwiener	<50	MIA/HIA	MIA/HIA/MIF/HIF/LIA	7	9th
	50–60				

DsFKuhawara	<50	**LIA**	**MIA/LIF/HIF**	**6**	**2nd **
	50–60		**LIA/MIF**		

DsFlsminsc	<50	LIA/HIF	HIF	5	4th
	50–60		LIA/MIA		

DsFmedian	<50		MIA/LIF/HIF	4	6th
	50–60		LIA		

DsFhybridmedian	<50	**LIA**	**MIA/LIF/HIF**	**7**	**1st **
	50–60		**LIA/LIF/MIF**		

DsFnldif	<50	LIA/MIA/LIF	MIA/HIA	9	3rd
	50–60		LIA/MIA/HIA/HIF		

DsFsrad	<50	LIA/HIF	MIA/LIF	7	5th
	50–60		LIA/MIA/HIA		

LIA, MIA, HIA: Low, Medium, High instantaneous amplitude. LIF, MIF, HIF: Low, medium, high instantaneous frequency, Score: Illustrates the numbers of significantly different features.

**Table 4:** Comparison of the mean, standard deviation (STD), median, and different quartile ranges between the high, medium and low AM-FM features extracted from the IMC for the three different age groups, below 50 (<50), between 50 and 60 (50–60) and above 60 (>60) years old for the original, the DsFhybrimedian and the DsFkuhawara filters. Here, the IA and IF values have been pre-multiplied by 100 for better visualization. Recall that the original images were normalized to a maximum brightness value of 1. Thus, the IA values represent a percentage of the maximum input image intensity. The instantaneous frequency magnitude, IF, is measured in cycles/mm (100x, Magnified by 100).

		Mean	STD	Median	P5%	P10%	P25%	P75%	P90%	P95%
Original	LIA: <50	2.47	0.45	2.29	1.81	1.9	2.2	2.8	3.0	3.4
	LIA: 50–60	2.88	0.66	2.62	2.31	2.5	2.5	2.7	4.0	4.3
	LIA: >60	2.6	**0.42**	2.48	1.91	2.26	2.33	2.81	3.33	3.35
	LIF: <50	145	4.1	145	140	141	142	146	147	154
	LIF: >60	144	**5.2**	144	135	137	139	145	150	153
	LIF: 50–60	143	**3.8**	145	136	137	139	146	147	147
	MIF: 50–60	285	8.0	284	275	276	280	289	296	303
	MIF: >60	284	8.7	282	273	275	278	289	295	303
	HIF: <50	574	**2.8**	574	545	546	556	578	599	641
	HIF: >60	566	**1.6**	564	541	545	557	574	585	597

DsFhybrimedian	LIA: <50	2.5	0.44	2.3	1.88	2.06	2.19	2.78	3.03	3.42
	LIA: 50–60	2.64	0.64	2.64	2.32	2.36	2.45	2.78	3.92	4.33
	LIA: >60	1.95	**0.37**	1.86	1.47	1.52	1.68	2.16	2.58	2.75
	LIF: <50	146	3.89	146	142	143	144	149	150	155
	LIF: >60	144	4.71	143	137	138	141	148	151	152
	LIF: 50–60	143	**5.82**	146	136	137	141	147	147	148
	MIF: 50–60	284	8.22	283	273	274	279	287	296	301
	MIF: >60	283	8.35	281	274	275	276	290	296	302
	HIF: <50	564	**17.76**	568	539	541	550	570	586	599
	HIF: >60	556	**12.6**	553	540	543	545	566	575	580

DsFKuhawara	LIA: <50	2.5	0.44	2.32	1.89	2.07	2.18	2.77	3.03	3.41
	LIA: 50–60	2.63	0.62	2.61	2.3	2.37	2.41	2.79	3.91	4.32
	LIA: >60	2.58	0.39	2.49	1.92	2.27	2.32	2.77	3.24	3.41
	LIF: <50	146	3.89	146	142	142	144	148	149	155
	LIF: >60	144	4.71	143	137	138	141	148	151	152
	LIF: 50–60	144	3.82	146	136	137	141	147	148	149
	MIF: 50–60	284	8.22	283	273	274	279	287	296	301
	MIF: >60	283	8.3	281	274	275	276	290	296	303
	HIF: <50	564	**17.8**	568	539	541	550	570	586	599
	HIF: >60	556	**12.5**	553	540	543	546	566	575	580

IMC: Intima-media-complex.
